# Effects of Chronic Tinnitus on Metabolic and Structural Changes in Subjects With Mild Cognitive Impairment

**DOI:** 10.3389/fnagi.2020.594282

**Published:** 2020-11-19

**Authors:** Sang-Yeon Lee, Heejung Kim, Jun Young Lee, Ju Hye Kim, Dong Young Lee, Inhee Mook-Jung, Young Ho Kim, Yu Kyeong Kim

**Affiliations:** ^1^Department of Otorhinolaryngology, Seoul National University Bundang Hospital, Seoul National University College of Medicine, Seoul, South Korea; ^2^Department of Nuclear Medicine, Seoul National University Boramae Medical Center, Seoul National University College of Medicine, Seoul, South Korea; ^3^Institute of Radiation Medicine, Medical Research Center, Seoul National University College of Medicine, Seoul, South Korea; ^4^Department of Psychiatry, Seoul National University Boramae Medical Center, Seoul National University College of Medicine, Seoul, South Korea; ^5^Neuroscience Research Institute, Seoul National University College of Medicine, Seoul, South Korea; ^6^Departmentof Psychiatry, Seoul National University College of Medicine, Seoul, South Korea; ^7^Institute of Human Behavioral Medicine, Medical Research Center, Seoul National University, Seoul, South Korea; ^8^Department of Biochemistry and Biomedical Science, Seoul National University College of Medicine, Seoul, South Korea; ^9^Department of Otorhinolaryngology, Seoul National University Boramae Medical Center, Seoul National University College of Medicine, Seoul, South Korea

**Keywords:** tinnitus, mild cognitive impairment, voxel-based morphometry, positron emission tomography, Alzheimer’s dementia

## Abstract

Tinnitus is a conscious auditory perception in the absence of an external stimulus. Despite previous reports of a recognized association between tinnitus and cognitive deficits, the effects of tinnitus on functional and structural brain changes associated with cognitive deficits remain unknown. We aimed to investigate the changes in glucose metabolism and gray matter (GM) volume in subjects diagnosed with mild cognitive impairment (MCI) depending on tinnitus. Twenty-three subjects were subclassified into MCI with the chronic tinnitus (MCI_T) and MCI without tinnitus (MCI_NT) groups. Encouraged by the identification of neural substrates associated with tinnitus and cognitive deficits, we correlated the extent of tinnitus severity with the changes in glucose metabolism and GM volume and conducted a glucose metabolic connectivity study. Compared to the MCI_NT group, the MCI_T group showed significantly lower metabolism in the right superior temporal pole and left fusiform gyrus. Additionally, the GM volume in the right insula was markedly lower in the MCI_T group compared to the MCI_NT group. Moreover, correlation analyses in metabolism or GM volumes revealed specific brain regions associated with the cognitive decline with increasing tinnitus severity. Metabolic connectivity analysis revealed that MCI_NT had markedly strengthened intra-hemispheric connectivity in the frontal, parietal, and occipital regions than did MCI_T. Furthermore, MCI_NT showed a strong negative association between the parietal and temporal and parietal and limbic regions, but the association was not observed in MCI_T. These findings indicate that tinnitus may cause metabolic and structural changes in the brain and alters complex inter- or intra-hemispheric networks in MCI. Considering the impact of MCI on accelerating dementia, these results provide a valuable basis on which yet-to-be-identified neurodegenerative markers of tinnitus can be refined.

## Introduction

Tinnitus, a “phantom sound,” is a conscious auditory perception in the absence of an external stimulus (Lee et al., 2017). Recently, tinnitus is considered to be a consequence of the complex interplay between auditory and non-auditory cortical regions after auditory deafferentation, likely recapitulating maladaptive cortical plasticity (Langguth et al., 2013). A meta-analysis of PET studies, coupled with other neuroimaging-based researches, has shown an association between tinnitus and multiple brain regions concerning attention, emotion, memory, and cognition (Song et al., 2012).

With a growing body of evidence on the association between chronic tinnitus and cognitive deficits, several studies have suggested that a decrease in attention and working memory is associated with the mechanism between chronic tinnitus and cognitive deficits (Rossiter et al., 2006; Trevis et al., 2016; Zarenoe et al., 2017). Furthermore, by correlating resting-state cortical oscillatory changes with tinnitus severity, a recent study has proposed that specific brain regions related to memory, such as the parahippocampus, may serve as a bridge between chronic tinnitus and cognitive decline. This, in turn, led us to hypothesize that neurophysiological changes may explain the association between tinnitus and cognitive impairment.

Specifically, mild cognitive impairment (MCI) is a predementia condition with a substantial risk of advancing to dementia (Snowden, 2004), particularly Alzheimer’s disease (Levey et al., 2006; Langa and Levine, 2014). Therefore, evaluating the risk factors associated with MCI is important for prognosis and protection. It was recently observed that chronic tinnitus accompanies a relatively high rate of MCI in approximately 17% of elderly subjects (i.e., >65 years; Lee et al., 2020). Further, a significant correlation between tinnitus severity and cognitive performance suggests that chronic tinnitus might be a potential determinant for accelerating MCI (Wang et al., 2018; Lee et al., 2020). The rationale behind this association would rely on a couple of previous studies, demonstrating that tinnitus may closely link to a reduced cognitive function on selective and divided attention, memory, and learning (Das et al., 2012; Vanneste et al., 2016). Despite the existence of recognized evidence regarding the association between tinnitus and cognitive deficits, the effects of chronic tinnitus on functional and structural brain changes in subjects with MCI have never been investigated, and no neurodegenerative markers of chronic tinnitus have thus far been identified.

Herein, we thus aimed to investigate the changes in glucose metabolism and gray matter (GM) volume in subjects diagnosed with MCI depending on tinnitus using [^18^F]fluoro-2-deoxyglucose-positron emission tomography (FDG-PET) and voxel-based morphometry (VBM). A recent neuroimaging study demonstrated that the combination of FDG-PET and VBM makes it possible to predict the conversion from MCI to Alzheimer’s dementia (Ottoy et al., 2019). Encouraged by the identification of neural substrates associated with tinnitus and cognitive deficits, we correlated the extent of tinnitus severity with the changes in glucose metabolism and GM volume. Furthermore, we conducted a glucose metabolic connectivity study based on the correlation of FDG uptakes between predefined regions of interest by templates to reveal the specific tinnitus-related metabolic pattern in the MCI group, similar to functional reorganization. Certainly, the functional connectivity analyses based on FDG-PET have been developed (Yakushev et al., 2017), allowing to evaluate cerebral metabolic connectivity using inter-regional correlation analysis (Lee et al., 2008). Overall, the present study not only provides insights regarding the effects of chronic tinnitus on metabolic and structural changes in patients with MCI but also sets the stage for potential neural substrates that may link tinnitus and cognitive decline.

## Materials and Methods

### Subjects

This study retrospectively reviewed subjects diagnosed with MCI who were nested in the prospective, longitudinal cohort registry of the Korean Brain Aging Study for the Early Diagnosis and Prediction of Alzheimer’s disease. Only subjects whose baseline neuroimaging was performed and audiograms met the criteria of having a mean hearing threshold <40 dB hearing loss (HL) in both ears were initially included. Subsequently, subjects with otologic disorders such as otosclerosis and Meniere’s disease, psychiatric or neurological disorders, and chronic headache, subjects receiving psychotropic/central nervous system-active medications, and subjects with a history of drug/alcohol abuse and/or history of a head injury (with loss of consciousness) or seizures were excluded from this study. Ultimately, 23 eligible subjects were enrolled in this study. To test the hypothesis, 23 subjects were subclassified into two groups: MCI with chronic subjective tinnitus (the MCI_T group, *N* = 12) and MCI without chronic subjective tinnitus (the MCI_NT group, *N* = 11). All subjects in the MCI_T group experienced perception of tinnitus with a duration of more than 6 months. Specifically, two subjects who reported no subjective tinnitus but had positive Tinnitus Handicap Inventory (THI) scores of 5 or less were assigned to the MCI_NT group. This study was approved by the Seoul National University Hospital Institutional Review Board (IRB-B-20-2019-44) and was conducted following the Declaration of Helsinki.

### Mild Cognitive Impairment Criteria and Neurocognition Battery

All subjects were diagnosed with MCI based on Peterson criteria, as documented in a previous study (Byun et al., 2017). The subjects had a global Clinical Dementia Rating score of 0.5 and were assessed at baseline and follow-up according to the Korean version of Consortium to Establish a Registry for Alzheimer’s Disease (CERAD-K) neuropsychological battery (Lee et al., 2004). The CERAD-K neuropsychological battery comprised the Verbal Fluency Test, Boston Naming Test, Mini-Mental State Exam in the Korean version of the CERAD assessment packet, Word List Memory, Constructional Praxis, Word List Recall, Word List Recognition, and Constructional Recall Test. General exclusion criteria for all patients were a history of major neurological or untreated major medical conditions.

### Positron Emission Tomography (PET)/Magnetic Resonance Image Acquisition

Subjects underwent FDG-PET and magnetic resonance (MR) imaging using a PET/MR scanner (Biograph mMR, Siemens Healthcare, Knoxville, TN, USA). Subjects received an intravenous injection of 370 MBq or less of [^18^F] FDG, the subjects remained in a dimly lit waiting room, and the brain emission scans were acquired after 40 min on bolus injection and continued for 20 min. For attenuation correction of PET, MR images were acquired simultaneously with PET using a dual-echo ultrashort echo time (UTE) sequence (echo time = 0.07 and 2.46 ms, repetition time = 11.9 ms, flip angle = 10°). The UTE images were reconstructed into a 192 × 192 × 192 matrix with an isotropic voxel size of 1.33 mm. The PET images were reconstructed using the ordered subset expectation maximization algorithm (subset = 21, iteration = 6) into 344 × 344 × 127 matrices with voxel size 1.04 × 1.04 × 2.03 mm. A 6-mm Gaussian post-filter was applied to the reconstructed PET images. A T1-weighted three-dimensional ultrafast gradient echo sequence was also acquired on an integrated PET/MR scanner in a 208 × 256 × 256 matrix with voxel sizes of 1.0 × 0.98 × 0.98 mm.

### Audiological and Psychoacoustic Evaluations

At the baseline evaluation, a structured history of the characteristics of tinnitus on the affected ear and the psychoacoustic properties (pure-tone or narrow-band noise) of the tinnitus was obtained. As described in previous studies (Lee et al., 2017, 2020), all subjects underwent pure-tone audiometry (PTA) testing that included psychoacoustic tests of tinnitus such as tinnitus pitch matching, tinnitus loudness matching, and the minimum masking level test. The hearing thresholds for seven different octave frequencies (0.25, 0.5, 1, 2, 3, 4, and 8 kHz) were evaluated using PTA in a sound-proof booth. The mean hearing threshold was calculated by the average of the hearing thresholds at 0.5, 1, and 2 kHz. The severity of perceived tinnitus was based on the THI scores.

### PET Analysis

Pre-processing and statistical analyses were performed using Statistical Parametric Mapping (SPM12, Wellcome Department of Imaging Neuroscience, London, UK^1^) implemented in MATLAB 9.1 (The MathWorks Inc., Natick, MA, USA). Co-registration was performed to align functional and structural images from the same subject to map functional information into anatomical space, and the co-registered FDG images were subsequently spatially transformed into the Montreal Neurological Institute standard PET template. The spatially normalized image was smoothed with an isotropic Gaussian kernel of 12 mm full width at half maximum (FWHM). Brain glucose metabolism at each voxel was proportionally scaled to the global mean value to reduce individual variation; hence, the relative regional glucose metabolic rate was calculated.

### Voxel-Based Morphometry Image Analyses

VBM was performed using the CAT12 toolbox^2^; Structural Brain Mapping Group, Jena University Hospital, Jena, Germany) implemented in SPM12 to identify structural changes. Each anatomical image was segmented into GM, white matter, and cerebrospinal fluid and non-linearly normalized to a standard stereotactic space using DARTEL (diffeomorphic anatomical registration through an exponentiated Lie algebra) algorithm. The spatially normalized images were subsequently rescaled to preserve relative tissue volumes and smoothed using an 8-mm FWHM Gaussian kernel to reduce residual interindividual variability. For the exclusion of artifacts on the GM, we applied an absolute GM threshold of 0.1.

### FDG-PET Metabolic Connectivity

For whole-brain FDG-PET metabolic connectivity, we used a region of interest (ROI)-based metabolic connectivity. First, the pre-processed and normalized FDG-PET image for subjects was parcellated based on the Automated Anatomical Labeling (AAL) template, which divides the brain into 90 anatomical ROIs, except the cerebellum (Supplementary Table 1, Tzourio-Mazoyer et al., 2002). To evaluate FDG-PET metabolic connectivity, we extracted the count normalized mean glucose uptake values divided by global mean from each ROI of the AAL template for all subjects, calculated the Pearson’s correlation between each pair of ROIs across subjects within each group, and created a pairwise FDG-PET metabolic connectivity matrix (90 × 90 ROIs) for the whole brain in each group. Age was included as a nuisance variable. A connectivity matrix was constructed by converting the correlation coefficient values into Fisher’s *Z* values to obtain an approximately normal distribution. Moreover, individual FDG-PET metabolic connectivity maps were constructed for each group separately at a threshold of *p* < 0.01, and the difference in each real FDG-PET metabolic connectivity matrix between the two groups (e.g., MCI_T vs. MCI_NT) was also constructed with significance at a *p* < 0.01 (two-tailed). For validation, we performed nonparametric permutation testing to test the probability that the observed difference of metabolic connectivity between the two groups occurred by chance (the null hypothesis) and to validate significant differences. To determine the null distribution of the difference in the correlation between the determined ROIs, we went back to the data matrix. The subject number was randomly into two pseudo-group data matrices (e.g., pseudo-MCI_T and pseudo-MCI_NT). On each pseudo-data matrix, correlation matrices were generated, and differences between two pseudo-groups were computed. Subsequently, null distributions of the FDG-PET metabolic connectivity matrix were generated, and this procedure was repeated 10,000 times. The difference in each real FDG-PET metabolic connectivity matrix between the two groups was compared with the null distribution. Significance was set at a *p* < 0.01 (two-tailed).

### Statistical Analysis

To evaluate differences between the MCI_T and MCI_NT groups in glucose metabolism and gray matter volume, statistical tests were performed using a two-sample *t*-test. The age variable was included as a nuisance covariate for glucose metabolism differences and age and total intracranial volume (TIV) were included as covariates of no interest for differences of gray matter volume. Because of the small sample size, the statistical voxel-wise threshold was set at uncorrected *p* < 0.005 with a cluster extent threshold of 100 in group comparison. For correlation analysis, after a log conversion of the THI scores due to the wide range (from 0 to 88), an evaluation of the correlation between the severity of tinnitus based on THI scores and gray matter volume and between the severity of tinnitus based on THI scores and glucose metabolism were also evaluated using regression analysis for whole-brain volume and glucose metabolism. The age variable was included as a nuisance covariate in regression analysis in glucose metabolism and the TIV variable was added in regression analysis in gray matter volume. For regression analysis, an exploratory uncorrected statistical threshold was set at *p* < 0.005 and a minimum cluster extent of 100 voxels in regression analysis in gray matter volume and a minimum cluster extent of 50 voxels in regression analysis in glucose metabolism. For validation of metabolic connectivity analysis, we performed non-parametric permutation testing by 10,000 times. The difference in metabolic connectivity matrix between two groups was set at a *p* < 0.01 (two-tailed).

## Results

### Demographic and Clinical Characteristics of the Subjects

**Figure 1:**
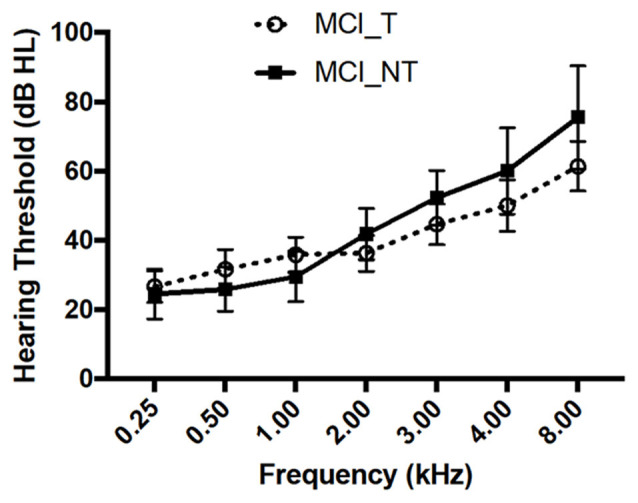
No significant differences in hearing thresholds across all frequencies were observed between mild cognitive impairment (MCI) with tinnitus handicap (MCI_T) and MCI without tinnitus handicap (MCI_NT). Data show the mean ± SEM (SEM, standard error of the mean).

The clinical characteristics of 23 subjects diagnosed with MCI are summarised in Table 1. The mean age of the 23 subjects was 74.0 ± 6.1 years (range, 63–83 years), and 13 were male. The demographics and clinical characteristics in terms of age, sex, educational level, and mean hearing thresholds of subjects in the MCI_T and MCI_NT groups did not have a statistically significant difference. In particular, each hearing threshold across all frequencies did not differ between the MCI_T and MCI_NT groups (Figure 1). Regarding the neuropsychological test, no significant differences were observed for any domain involved in CERAD-K between the two groups. As expected, THI scores and the duration of tinnitus were significantly higher in the MCI_T group than those in the MCI_NT group. In the MCI_T group (*N* = 12), the most frequent characteristic of tinnitus was pure tone (*N* = 7, 58.3%), followed by narrow-band noise (*N* = 5, 41.7%).

**Table 1:** Demographic and clinical characteristics of the study population.

	MCI_T group (MCI patients with from mild, to severe tinnitus handicap)	MCI_NT group (MCI patients without tinnitus handicap)	*P*-value
No. of patients	12	11	
Gender (M:F)	6:6	7:4	
Age (years)	73.27 ± 5.83	74.83 ± 6.56	n.s
Education (years)	8.00 ± 4.43	11.82 ± 4.51	*p* = 0.053
Duration of tinnitus (years)	3.50 ± 3.19	0.09 ± 0.20	*p* < 0.01
THI	38.50 ± 20.06	0.55 ± 1.29	*p* < 0.01
Hearing loss (right)	32.71 ± 7.21	33.50 ± 8.42	n.s
Hearing loss (left)	29.69 ± 6.93	32.84 ± 6.94	n.s
Hearing loss (average)	31.20 ± 5.86	33.17 ± 7.22	n.s
Neuropsychological test			
MMSE	23.42 ± 2.75	24.73 ± 3.10	n.s
Semantic fluency	13.50 ± 3.66	11.64 ± 4.90	n.s
Boston naming	11.75 ± 1.60	11.64 ± 2.34	n.s
Word list immediate memory	15.33 ± 2.74	15.45 ± 3.67	n.s
Constructional praxis	9.50 ± 1.78	9.64 ± 1.21	n.s
Word list delayed recall	4.83 ± 1.19	4.00 ± 2.24	n.s
Word list recognition recall	8.75 ± 1.29	7.73 ± 2.53	n.s
Memory delayed call	6.08 ± 2.64	6.09 ± 3.33	n.s

*Data shows mean ± SD (SD, standard deviation). M, male; F, female; MMSE, mini mental state exam; THI, tinnitus handicap inventory; n.s, no statistical significance*.

### Group Comparison of Gray Matter Volume and Glucose Metabolism

**Figure 2:**
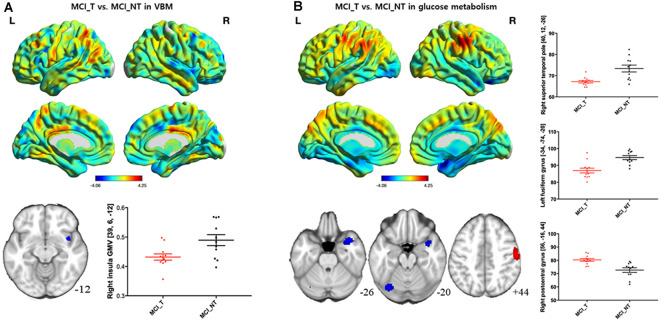
Brain regions showing structural and metabolic differences in MCI_T, compared with MCI_NT. **(A)** Three-dimensional visualization of comparison of MCI_T with MCI_NT in gray matter (GM) volumes by voxel-based morphometry (VBM). The red-spectrum color indicates the MCI_T-increase in GM volumes, and blue-spectrum color indicates the MCI_T-decrease in GM volumes compared with MCI_NT. MCI_T showed a significant decrease in GM volume, compared with MCI_NT (middle). The bottom figure shows a scatter plot of individual GM volume in the region showing significant volume reduction. The horizontal lines represent the mean and standard error of the mean (±SEM). **(B)** Three-dimensional visualization of comparison of MCI_T with MCI_NT in mean metabolic uptake of [^18^F]fluoro-2-deoxyglucose-positron emission tomography (FDG-PET). The red-spectrum color indicates the MCI_T-increase in metabolism, and blue-spectrum color indicates the MCI_T-decrease in metabolism compared with MCI_NT. Middle figures indicate brain regions showing significant glucose metabolic differences, in which red color indicates higher metabolism and blue color indicates lower metabolism in the MCI_T compared with the MCI_NT. The bottom figure shows a scatter plot of individual glucose metabolism in the regions showing significantly increased or decreased metabolic uptakes. The horizontal lines represent the mean and standard error of the mean (±SEM; *p* < 0.005 uncorrected, *k* > 100).

Compared with the MCI_NT group, the MCI_T group exhibited significantly lower GM volume in the right insula (Figure 2A; Table 2). Compared with the MCI_NT group, the MCI_T group showed a lower metabolism in the right superior temporal pole and the left fusiform gyrus and higher metabolism in the right postcentral gyrus (Figure 2B; Table 2).

**Table 2:** Brain regions showing significant gray matter (GM) volume or glucose metabolic differences between MCI_T and MCI_NT groups.

				Clusters		MNI Coordinates		
	Regions	L/R	BA	(voxels)	T-score	*x*	*y*	*z*
**Gray matter volumes differences**
MCI_T < MCI_NT	Insula	R	13	142	3.59	39	6	−12
**Glucose metabolic differences**
MCI_T > MCI_NT	Postcentral gyrus	R	4	305	4.25	56	−16	44
MCI_T < MCI_NT	Superior temporal pole	R	38	246	4.06	40	12	−26
	Fusiform gyrus/cerebellum	L	19	184	4.05	−34	−74	−20

*The statistical threshold was p < 0.005 (uncorrected) with cluster threshold of 100 voxels*.

### Association Between Tinnitus Severity and Gray Matter Volume and Metabolism

**Figure 3:**
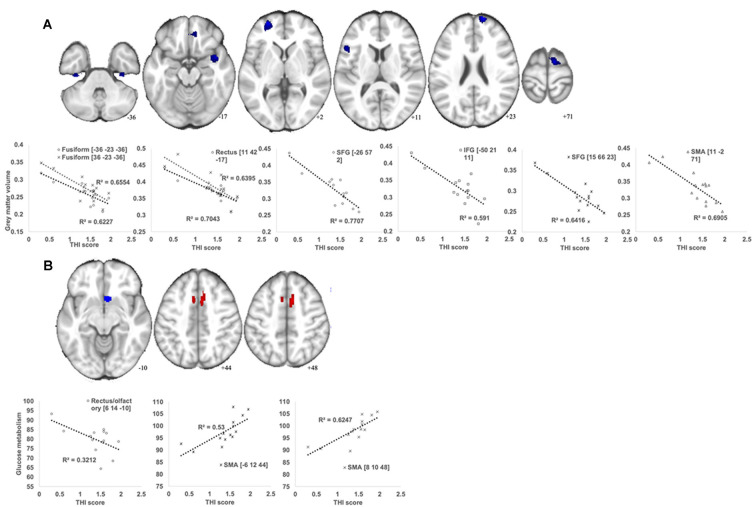
Brain regions showing significant correlations between the Tinnitus Handicap Inventory (THI) score and GM volume **(A)** and glucose metabolism **(B)** in the MCI_T group. **(A)** A voxel-wise multiple regression analysis was performed to detect specific areas in which the GM volume changes are associated with the THI score in MCI_T. The plot figure shows the linear regression line in each region. All analysis was controlled by age and total intracranial volume (TIV; *p* < 0.005 uncorrected, *k* > 100). **(B)** A voxel-wise multiple regression analysis was performed to detect specific areas in which the glucose metabolic changes are associated with the THI score in MCI_T. The plot figure shows the linear regression line in each region. All analysis was controlled by age (*p* < 0.005 uncorrected, *k* > 50).

The THI score was inversely correlated with the GM volume in multiple brain regions, including the bilateral superior frontal gyrus, left frontal gyrus, right supplementary motor area (SMA), right insula, bilateral fusiform gyrus, and right rectal gyrus (Figure 3A; Table 3). Additionally, a putative rank in terms of the T-score was observed. Specifically, the left superior frontal gyrus showed the highest correlation, whereas the SMA and insula belonged to the second-tier group. However, the THI score was positively associated with glucose metabolism in the SMA/middle cingulate gyrus but was inversely associated with that in the olfactory/rectal gyrus (Figure 3B; Table 3).

**Table 3:** Correlation analysis between glucose metabolism or gray matter volume and tinnitus severity in MCI_T group.

				Clusters		MNI Coordinates		
Regions		L/R	BA	(voxels)	T-score	*x*	*y*	*z*
**Gray matter volume correlation with tinnitus severity**
Negative correlation	Superior frontal gyrus	L	10	417	7.90	−26	57	2
	Superior frontal gyrus	R	10	228	7.15	15	66	23
	Inferior frontal gyrus	L	45	158	5.13	−50	21	11
	SMA	R	6	290	7.18	11	−2	71
	Insula	R	13	425	6.08	42	6	−17
	Fusiform gyrus	L	36	167	5.02	−36	−23	−36
	Fusiform gyrus	R	36	161	4.65	36	−23	−36
	Gyrus rectus	R	11	330	4.32	11	42	−17
**Glucose metabolism correlation with tinnitus severity**
Positive correlation	SMA/middle cingulate gyrus	R	6	201	4.81	8	10	48
	SMA/middle cingulate gyrus	L	6	61	3.73	−6	12	44
Negative correlation	Olfactory/ Gyrus rectus	R	32	95	3.59	6	14	−10

*The statistical threshold was p < 0.005 (uncorrected) with cluster threshold of 100 voxels (gray matter volume) and cluster threshold of 50 voxels (glucose metabolism)*.

### Metabolic Connectivity of Glucose Metabolism

**Figure 4:**
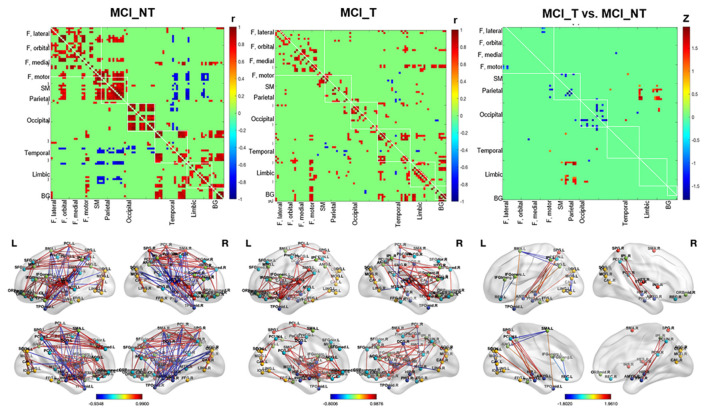
Whole-brain glucose metabolic connectivity. (First left and middle column) Figures show the whole-brain metabolic connectivity matrix among 90 by 90 regions of MCI_NT and MCI_T based on correlation, respectively, with color gradient representing the strength of correlation between two nodes, at *p*-value < 0.01. (Right column) The figure shows differences in metabolic connectivity in MCI_T compared to MCI_NT, at *p*-value < 0.01. Values were based on the z-transformed correlations within (diagonal) and between (off-diagonal) ROIs. The color bar represents Fisher’s Z. The difference in real connectivity matrix was compared with the null distribution, generated by permutation testing. The second row shows connections between two nodes projected on a 3D template.

In MCI_NT, negative metabolic connectivity was mainly detected between the parietal and temporal regions, such as Heschl’s gyrus, and between the parietal and limbic regions, including the amygdala, hippocampus, and parahippocampus. Moreover, MCI_NT showed strong metabolic connectivity within the intra-hemispheric regions, such as the frontal, parietal, and occipital regions, and between the frontoparietal regions (Figure 4). In contrast, in MCI_T, different from MCI_NT, inter-parietal connectivity was weakened or absent with the other regions, but there was strong connectivity between the motor regions and both temporal and limbic regions (Figure 4). Compared with the MCI_NT group, the MCI_T group had significantly lower metabolic connectivity between the rectal gyrus and inferior frontal gyrus; between the SMA and parietal region, including the angular gyrus and precuneus; between the orbitofrontal and inferior temporal region; between the precuneus and inferior occipital gyrus; and between the fusiform gyrus and insula but had higher metabolic connectivity between the parietal and temporal and parietal and limbic regions (Figure 4).

## Discussion

This is the first study that investigated the effects of chronic tinnitus on metabolic and structural brain changes concerning MCI. It clearly showed that at least chronic tinnitus contributes to hypometabolic changes in the fusiform gyrus and the superior temporal gyrus and volumetric atrophy in the insula. These results, coupled with correlation and connectivity analyses, merit attention because the specific brain regions tied to tinnitus and cognitive deficits may serve as neurodegenerative markers indicating the progression of cognitive deficits over time. However, the biomarkers that reveal the linkage between tinnitus and cognitive decline may be only relevant to the particular situation of MCI with tinnitus. Our findings cannot necessarily represent all cases of MCI transitioning dementia, requiring careful interpretation.

Importantly, the MCI_T group demonstrated more distinctive hypometabolic changes in the superior temporal pole and the fusiform gyrus compared to the MCI_NT group. Anatomically, the superior temporal region comprises the auditory cortex, which is known to play a critical role in tinnitus perception (Maudoux et al., 2012). As is known, the superior temporal region interacts with the parietal and limbic regions in the social cognitive process (Zilbovicius et al., 2006). A recent meta-analysis on PET studies reported higher regional cerebral blood flow in primary and secondary auditory cortices in tinnitus subjects compared with normal controls (Song et al., 2012). According to these results, the group with tinnitus should have high intrinsic activity or metabolism, but our result shows that metabolism in the tinnitus group is lower than that with the no tinnitus group. Possibly, the neurodegeneration might be still more advanced, despite an increase in intrinsic activity by tinnitus. This suggests that the hypometabolic changes in the superior temporal pole, as evidenced here, might be more associated with cognitive deficits and underlying tinnitus, rather than perceived tinnitus itself. Additionally, a recent study on changes in resting-state brain function networks in subjects with amnestic MCI showed that regional abnormalities in functional brain areas, including the superior temporal gyrus, could be associated with cognitive deficits (Wang et al., 2011). Several lines of evidence indicate that the superior temporal region plays a critical role in cognition. In particular, the superior temporal gyrus is an essential structure for auditory processing, which has been implicated as a hub for social perception and cognition (Ramot et al., 2019). For example, impaired social interaction and visual object discrimination deficiency, both early signs of MCI or Alzheimer’s dementia, are closely associated with the functional and structural abnormality of the superior temporal gyrus (Pietschnig et al., 2016; Ramot et al., 2019).

Additionally, compared with the MCI_NT group, a significant hypometabolism in the fusiform gyrus in the MCI_T group was observed. Furthermore, it shows that an increase in tinnitus severity is associated with the reduced GM volume of the fusiform gyrus. The fusiform gyrus is a part of the temporal lobe in Brodmann area 37, which has been associated with various neural pathways related to recognition (Mummery et al., 2000). A previous FDG-PET study revealed that the fusiform gyrus, but not the temporal pole, exerted a significant effect on semantic disruptions in semantic dementia (Cai et al., 2015). Moreover, the GM volume of the left fusiform gyrus was significantly correlated with the semantic scores in subjects with semantic dementia, after adjusting for the GM volumes of the other related regions (Ding et al., 2016). Collectively, these results suggest that perceived tinnitus in MCI subjects is closely associated with reduced activation of the superior temporal pole and fusiform gyrus. The specific brain regions tied to tinnitus and cognitive deficits, such as the superior temporal pole and fusiform gyrus, may act as potential neurodegenerative markers that may accelerate cognitive decline, which deserves further study.

Metabolic connectivity analysis revealed that, in MCI_NT, strong negative correlations were observed between the parietal regions and both temporal and limbic regions, which was less pronounced or absent in MCI_T. Negative correlations between a region and other brain areas indicate that when the region has a high FDG uptake, the negatively connected areas have a low FDG uptake and vice versa. In functional MRI (fMRI) network analyses, negative correlations between brain areas were frequently observed, but those had been considered artifacts caused by methodological peculiarities of fMRI analysis because their biological relevance was unclear (Parente et al., 2018). However, our results using FDG-PET were not influenced by short-term hemodynamics or time series artifacts. In recent studies, negative correlations tend to be considered in the biological state. Furthermore, negative correlations might reflect regulatory interactions between brain regions, such as modulations, inhibition, suppression, and neurofeedback (Gopinath et al., 2015). In the MCI_NT group, the inter-hemispheric parietal connectivity was negatively strengthened in temporal including Heschl’s gyrus and limbic regions, including the hippocampus, amygdala, and parahippocampal gyrus, although that was not observed in the MCI group with tinnitus. In particular, the MCI_T group showed hypermetabolism in the parietal regions. Our results suggested that hypermetabolism in the parietal regions was affected by chronic tinnitus in the MCI_T group, which was abnormally altered metabolic connectivity with several regions that impairs salience network and hearing, cognitive, and emotional processing.

It is worth noting that chronic tinnitus only leads to a decrease in the insula volume in subjects with MCI. As depicted in Figure 3, an inverse correlation between tinnitus severity and insula volume supports the putative association. The insula is responsible for emotion and sympathetic activation and, according to an integrative model of tinnitus (De Ridder et al., 2014), has been considered a critical node of the salience network in the context of tinnitus (Vanneste and De Ridder, 2012). The salience network is a distributed functional-anatomical network that supports emotion and cognition (Uddin, 2015). Importantly, the insula intensively connects with the medial temporal lobe and the posteromedial part of the parietal cortex, which are known biomarkers showing the accelerating conversion from MCI to Alzheimer dementia (Lee et al., 2002; Ferreira et al., 2017; Xu et al., 2019). The results were consistent with those of a previous meta-analysis, indicating that the precuneus and posterior cingulate cortex play a significant role in the transition from MCI to Alzheimer’s dementia (Ma et al., 2018). Indeed, Carpenter-Thompson et al. (2015a) also proposed that the posterior cingulate and insula may be associated with an early emotional reaction to develop tinnitus in both task and resting states. Further, the recruitment of more frontal regions makes it possible to better control their emotional response and exhibit altered connectivity in the default mode network (Carpenter-Thompson et al., 2015a). The precuneus and posterior cingulate cortex resided in the posteromedial part of the parietal cortex are core components of the default mode network (Huijbers et al., 2012), a distributed functional-anatomic network exhibiting a high rate of metabolism in subjects not focused on the outside world, and decreases in activity across a range of cognitive loads (Shulman et al., 1997; Raichle et al., 2001; Kim, 2010). Overall, the volumetric atrophy in the insula is an important morphological marker that selectively develops along with tinnitus in MCI patients and may contribute to the progressive cognitive decline by impairing the connectivity between the brain regions involved in the salience network.

Additionally, a significant inverse correlation between GM volume in the frontal gyrus and THI score was also distinct in MCI subjects. In line with this, Carpenter-Thompson et al. (2015b) demonstrated that individuals with lower tinnitus distress engaged frontal regions to a greater extent to better control their emotional response to affective sounds. Indeed, the frontal regions, such as the prefrontal cortex and orbitofrontal cortex, are considered key areas for the integration of sensory and emotional aspects of tinnitus and the modulation of autonomic physiological responses (Vanneste et al., 2010). Interestingly, the structural abnormalities of the frontal lobe have been reported to weaken the role of auditory memory storage, resulting in the inhibitory modulation of input to the auditory cortex (Voisin et al., 2006). Given this, severely attenuated GM volume in the frontal gyrus observed herein may represent the deficiency of auditory attention relevant to cognitive decline. As proposed in recent literature (Carpenter-Thompson et al., 2015b), changes in the function or structure of frontal gyrus might serve as a guide when evaluating the efficacy of tinnitus treatment for MCI subjects. Furthermore, it was observed that functional and structural changes in the SMA correlate with THI scores in subjects with MCI. Similarly, a recent study suggested that the conscious perception of tinnitus may be part of the synchronised theta activity in the SMA (Vanneste and De Ridder, 2012). Considering a potential link between the SMA and cognition (Nachev et al., 2008), hypermetabolic or diminished changes in the SMA, such as increasing tinnitus severity, may accelerate cognitive decline. Overall, these specific brain regions, yet-to-be-determined, may influence the progression of cognitive deficits over time, depending on the severity of tinnitus.

This milestone study merits special attention considering the significant impact of *chronic tinnitus* on developing dementia. These results enhance the understanding of the effects of chronic tinnitus on functional and structural brain changes in MCI subjects and offer some potential neurodegenerative markers indicative of cognitive decline. Nevertheless, several limitations require future follow-up investigations. First, our results are limited by the relatively small number of subjects in both groups, mainly due to the difficulty of recruiting MCI subjects with and without tinnitus presenting normal or mild hearing loss. Additionally, the current study was designed as a cross-sectional evaluation, which, along with the retrospective study design, may weaken the clinical implications of our results. Therefore, a prospective and longitudinal follow-up study in large-scale cases is required to support the hypotheses. Second, confounding variables concerning cognition were minimized, but not eliminated. Although only tinnitus subjects with normal hearing or mild hearing loss were enrolled, previous studies have noted that mild hearing loss still acts as a confounder that affects cognitive impairment, eventually leading to dementia (van Boxtel et al., 2000; Thomson et al., 2017). Moreover, combined tinnitus and hyperacusis were not taken into account. Tinnitus subjects may have different cortical activity patterns according to the degree of hearing loss or combined hyperacusis (Vanneste and De Ridder, 2016). Thus, more efforts to minimize the confounders that have cognition-related functional and structural brain changes are required to draw a firm conclusion. Third, lack of correction for multiple comparisons is also a major limitation of the study; future studies employing correction for multiple comparisons in large-scale cases would be the best fit for proving the effects of chronic tinnitus on functional and structural brain changes relevant to MCI. Fourth, since the average age is 74 and younger adults were not included, all results could be simply related to tinnitus, and not relevant to MCI or aging. All subjects in the present study were diagnosed with MCI based on Peterson criteria, which is consistent with the previous study in Korea (Byun et al., 2017). Also, the CERAD-K neuropsychological battery was employed to assess the psychometric properties of the various cognitive domains in both studies, including this study (Byun et al., 2017). As shown in Supplementary Table 2, compared with the “CN-old group by Byun et al. (2017), ” MCI group in this study markedly showed impairments of cognitive metrics in most cognitive metrics, except for Boston naming test and Memory delayed call. Nevertheless, the differences in neuropsychological status between the two groups were not completely adjusted by other confounding factors, such as age, gender, and education. Also, cognitive metrics were not rigorously included in the PET/MRI analyses. Thus, our results may still be inconclusive whether the effects of chronic tinnitus on functional and structural brain changes are relevant to MCI or the aging process or not, when considering additional potential confounders. Fifth, this study only included subjects with normal or mild hearing loss, raising a question that the relationships discovered herein might be different with higher levels of hearing loss. If not perfectly, previous studies could tell to some extent whether the relationships discovered herein will be different with higher levels of hearing loss or not. Hearing loss *per se* has been identified as the potentially largest modifiable risk factor for cognitive decline (Lin et al., 2011). Recent neuroimaging studies have shown that aberrant activity in the brain may interact with dementia pathology in people with hearing loss (Griffiths et al., 2020; Ha et al., 2020). Specifically, a longitudinal population study demonstrated that the risk of dementia increased with hearing loss severity for individuals older than 60 years (Lin et al., 2011). In collaboration with this, the worsening hearing was positively correlated with a higher β-amyloid burden, a pathologic biomarker of AD, measured *in vivo* with PET scans (Golub et al., 2020). Given this, the effects of chronic tinnitus on functional and structural brain changes in subjects with MCI would be different according to levels of hearing loss, but this awaits further confirmation. Nevertheless, we believe that our protocol that recruits only normal or mild hearing loss subjects exerts major strength because it can minimize bias related to hearing loss-induced metabolic and structural changes. Finally, subjects enrolled in this study show relatively heterogeneous tinnitus severity. To replicate current results, future studies comprising a large number of cases and subsequent normal distribution of tinnitus severity should be considered.

## Conclusions

Taken together, these results show, for the first time, that chronic tinnitus elicits differential metabolic and structural brain changes in subjects diagnosed with MCI. Given the significant impact of MCI on developing dementia, specifically Alzheimer’s disease, this study merits strong attention because the results provide a valuable basis on which neurodegenerative markers of tinnitus, yet-to-be-identified, can be polished accordingly.

## Data Availability Statement

The raw data supporting the conclusions of this article will be made available by the authors, without undue reservation.

## Ethics Statement

The studies involving human participants were reviewed and approved by the Seoul National University Hospital Institutional Review Board (IRB-B-20-2019-44) and was conducted in accordance with the Declaration of Helsinki. The patients/participants provided their written informed consent to participate in this study.

## Author Contributions

S-YL, YHK, and YKK: conceptualization. S-YL, HK, and YKK: methodology. JL and JK: software. YHK and YKK: validation and supervision. S-YL and HK: formal analysis, investigation and writing—original draft preparation. YKK: resources and funding acquisition. S-YL, HK, DL, JK, and JL: data curation. DL and I-MJ: writing—review and editing. HK: visualization. S-YL: project administration. All authors contributed to the article and approved the submitted version.

## Conflict of Interest

The authors declare that the research was conducted in the absence of any commercial or financial relationships that could be construed as a potential conflict of interest.

## Footnotes

^1^http://www.fil.ion.ucl.ac.uk/spm

^2^http://www.neuro.uni-jena.de/cat
