# Supplementary material for: Effects of Chronic Tinnitus on Metabolic and Structural Changes in Subjects With Mild Cognitive Impairment
Source: Front Aging Neurosci. 2020 Nov 19;12:594282. doi: 10.3389/fnagi.2020.594282 (PMC7710517; doi:10.3389/fnagi.2020.594282)
Supplement: Supplementary file 1 [file Table_1.docx]

Supplementary. Table 1. Whole-brain regions by the Automated Anatomical Labelling (AAL) template

| **Subnetwork** | **Regions** | **MNI coordinates** | **Subnetwork** | **Regions** | **MNI coordinates** |
| --- | --- | --- | --- | --- | --- |
| Frontal, lateral | Frontal_Inf_Oper_L | [-47, 10, 13] | Occipital | Calcarine_L | [-9, -81, 3] |
| Frontal, lateral | Frontal_Inf_Oper_R | [48, 14, 18] | Occipital | Calcarine_R | [13, -76, 7] |
| Frontal, lateral | Frontal_Inf_Tri_L | [-45, 28, 9] | Occipital | Cuneus_L | [-9, -81, 24] |
| Frontal, lateral | Frontal_Inf_Tri_R | [46, 30, 11] | Occipital | Cuneus_R | [10, -80, 26] |
| Frontal, lateral | Frontal_Mid_L | [-35, 29, 33] | Occipital | Fusiform_L | [-34, -44, -22] |
| Frontal, lateral | Frontal_Mid_R | [36, 32, 34] | Occipital | Fusiform_R | [33, -42, -21] |
| Frontal, lateral | Frontal_Sup_L | [-19, 33, 40] | Occipital | Lingual_L | [-16, -70, -8] |
| Frontal, lateral | Frontal_Sup_R | [19, 31, 42] | Occipital | Lingual_R | [15, -70, -7] |
| Frontal, orbital | Frontal_Inf_Orb_L | [-35, 28, -15] | Occipital | Occipital_Inf_L | [-38, -79, -10] |
| Frontal, orbital | Frontal_Inf_Orb_R | [37, 31, -14] | Occipital | Occipital_Inf_R | [36, -83, -7] |
| Frontal, orbital | Frontal_Med_Orb_L | [-7, 51, -11] | Occipital | Occipital_Mid_L | [-34, -82, 15] |
| Frontal, orbital | Frontal_Med_Orb_R | [5, 49, -10] | Occipital | Occipital_Mid_R | [35, -80, 19] |
| Frontal, orbital | Frontal_Mid_Orb_L | [-31, 47, -11] | Occipital | Occipital_Sup_L | [-19, -85, 26] |
| Frontal, orbital | Frontal_Mid_Orb_R | [29, 52, -12] | Occipital | Occipital_Sup_R | [21, -82, 28] |
| Frontal, orbital | Frontal_Sup_Orb_L | [-18, 44, -15] | Temporal | Heschl_L | [-43, -22, 8] |
| Frontal, orbital | Frontal_Sup_Orb_R | [15, 45, -16] | Temporal | Heschl_R | [43, -20, 9] |
| Frontal, medial | Frontal_Sup_Medial_L | [-7, 47, 29] | Temporal | Rolandic_Oper_L | [-47, -12, 13] |
| Frontal, medial | Frontal_Sup_Medial_R | [6, 49, 28] | Temporal | Rolandic_Oper_R | [49, -7, 13] |
| Frontal, medial | Olfactory_L | [-9, 12, -12] | Temporal | Temporal_Inf_L | [-50, -30, -26] |
| Frontal, medial | Olfactory_R | [7, 12, -12] | Temporal | Temporal_Inf_R | [51, -33, -23] |
| Frontal, medial | Rectus_L | [-7, 33, -21] | Temporal | Temporal_Mid_L | [-56, -36, -5] |
| Frontal, medial | Rectus_R | [5, 32, -21 | Temporal | Temporal_Mid_R | [55, -38, -2] |
| Frontal, motor | Precentral_L | [-39, -7, 48] | Temporal | Temporal_Pole_Mid_L | [-39, 9, -38] |
| Frontal, motor | Precentral_R | [38, -7, 50] | Temporal | Temporal_Pole_Mid_R | [41, 10, -35] |
| Frontal, motor | Supp_Motor_Area_L | [-7, 4, 59] | Temporal | Temporal_Pole_Sup_L | [-41, 11, -23] |
| Frontal, motor | Supp_Motor_Area_R | [7, 1, 58] | Temporal | Temporal_Pole_Sup_R | [44, 11, -19] |
| Somatosensory | Paracentral_Lobule_L | [-8, -25, 66] | Temporal | Temporal_Sup_L | [-54, -23, 5] |
| Somatosensory | Paracentral_Lobule_R | [7, -31, 65] | Temporal | Temporal_Sup_R | [55, -23, 6] |
| Somatosensory | Postcentral_L | [-43, -24, 48] | Temporal | Insula_L | [-36, 2, 1] |
| Somatosensory | Postcentral_R | [40, -23, 51] | Temporal | Insula_R | [36, 3, 1] |
| Parietal | Angular_L | [-44, -63, 35] | Limbic | Amygdala_L | [-25, -5, -19] |
| Parietal | Angular_R | [43, -60, 37] | Limbic | Amygdala_R | [24, -4, -20] |
| Parietal | Parietal_Inf_L | [-43, -48, 45] | Limbic | Cingulum_Ant_L | [-5, 33, 11] |
| Parietal | Parietal_Inf_R | [45, -46, 48] | Limbic | Cingulum_Ant_R | [7, 35, 15] |
| Parietal | Parietal_Sup_L | [-24, -58, 57] | Limbic | Cingulum_Mid_L | [-7, -16, 38] |
| Parietal | Parietal_Sup_R | [24, -57, 59] | Limbic | Cingulum_Mid_R | [7, -9, 37] |
| Parietal | Precuneus_L | [-8, -56, 46] | Limbic | Cingulum_Post_L | [-6, -46, 20] |
| Parietal | Precuneus_R | [8, -55, 41] | Limbic | Cingulum_Post_R | [6, -45, 17] |
| Parietal | SupraMarginal_L | [-57, -37, 28] | Limbic | Hippocampus_L | [-25, -24, -12] |
| Parietal | SupraMarginal_R | [56, -31, 35] | Limbic | Hippocampus_R | [27, -23, -12] |
| Basal ganglia | Caudate_L | [-13, 9, 8] | Limbic | ParaHippocampal_L | [-24, -20, -23] |
| Basal ganglia | Caudate_R | [13, 10, 9] | Limbic | ParaHippocampal_R | [24, -19, -23] |
| Basal ganglia | Pallidum_L | [-20, -2, -2] | Limbic | Thalamus_L | [-12, -20, 7] |
| Basal ganglia | Pallidum_R | [20, -2, -2] | Limbic | Thalamus_R | [12, -19, 7] |
| Basal ganglia | Putamen_L | [-25, 1, 1] |  |  |  |
| Basal ganglia | Putamen_R | [25, 2, 0] |  |  |  |
