# Supplementary material for: Effects of Chronic Tinnitus on Metabolic and Structural Changes in Subjects With Mild Cognitive Impairment
Source: Front Aging Neurosci. 2020 Nov 19;12:594282. doi: 10.3389/fnagi.2020.594282 (PMC7710517; doi:10.3389/fnagi.2020.594282)
Supplement: Supplementary file 2 [file Table_2.docx]

**Supplement Table 2. Comparison of cognitive metrics in a subset of neuropsychological tests**

|  | MCI group (N=23)  (by original cohort) | CN-old group (N=291)  (by Byun et al. 2017) | P-value |
| --- | --- | --- | --- |
| MMSE | 24.04 ± 2.93 | 26.90 ± 2.61 | P<0.001* |
| Semantic fluency | 12.61 ± 4.30 | 15.69 ± 4.77 | P=0.003* |
| Boston naming test | 11.70 ± 1.94 | 12.14 ± 2.19 | P=0.351 |
| Word list:  Immediate recall | 15.39 ± 3.14 | 19.45 ± 4.16 | P<0.001* |
| Word list:  Delayed recall | 4.44 ± 1.78 | 9.11 ± 1.22 | P <0.001* |
| Word list:  Recognition recall | 8.26 ± 2.01 | 10.07 ± 1.33 | P < 0.001* |
| Constructional Praxis | 9.57 ± 1.50 | 6.53 ± 1.85 | P < 0.001* |
| Memory delayed call | 6.09 ± 2.92 | 7.41 ± 2.86 | P=0.066 |
| Stroop test:  Word reading | 64.83 ± 16.66 | 76.96 ± 18.41 | P=0.014* |
| Stroop test:  Color reading | 55.35 ± 12.22 | 62.54 ± 13.27 | P=0.037* |
| Stroop test:  Color word-reading | 31.17 ± 11.12 | 37.70 ± 11.10 | P=0.028* |

MMSE, Mini-Mental State Examination; MCI, mild cognitive impairment; CN-old: Cognitively normal old-aged adults (Average age: 69.2 ± 8.1, Range: 55–87)
